# Identification of biomarkers associated with ferroptosis in diabetic retinopathy based on WGCNA and machine learning

**DOI:** 10.3389/fgene.2024.1376771

**Published:** 2024-05-28

**Authors:** Hui-qing Guo, Rong Xue, Guangming Wan

**Affiliations:** Department of Ophthalmology, First Affiliated Hospital of Zhengzhou University, Henan Province Eye Hospital, Zhengzhou, China

**Keywords:** diabetic retinopathy, ferroptosis, biomarker, WGCNA, machine learning

## Abstract

**Objective: **Diabetic retinopathy (DR) is a chronic progressive eye disease that affects millions of diabetic patients worldwide, and ferroptosis may contribute to the underlying mechanisms of DR. The main objective of this work is to explore key genes associated with ferroptosis in DR and to determine their feasibility as diagnostic markers.

**Methods: **WGCNA identify the most relevant signature modules in DR. Machine learning methods were used to de-screen the feature genes. ssGSEA calculated the scoring of immune cells in the DR versus control samples and compared the associations with the core genes by Spearman correlation.

**Results: **We identified 2,897 differential genes in DR versus normal samples. WGCNA found tan module to have the highest correlation with DR patients. Finally, 20 intersecting genes were obtained from differential genes, tan module and iron death genes, which were screened by LASSO and SVM-RFE method, and together identified 6 genes as potential diagnostic markers. qPCR verified the expression and ROC curves confirmed the diagnostic accuracy of the 6 genes. In addition, our ssGSEA scoring identified these 6 core genes as closely associated with immune infiltrating cells.

**Conclusion: **In conclusion, we analyzed for the first time the potential link of iron death in the pathogenesis of DR. This has important implications for future studies of iron death-mediated pro-inflammatory immune mechanisms.

## 1 Introduction

Diabetic retinopathy (DR) is a chronic and progressive eye disease that affects millions of people with diabetes worldwide. It is caused by damage to the blood vessels of the retina, the light-sensitive tissue at the back of the eye, due to high blood sugar levels. DR can lead to vision loss and blindness if left untreated, and is associated with increased risk of other complications such as cardiovascular disease, kidney failure, and neuropathy (Cheung and Wong, 2008; Cheung et al., 2010). Therefore, early diagnosis and timely treatment of DR are essential to prevent or delay its devastating consequences.

High-throughput sequencing (HTS) is a powerful technology that enables the analysis of the genome, transcriptome, epigenome, and microbiome of biological samples at an unprecedented scale and speed (Singer et al., 1992). HTS combined with comprehensive bioinformatics analysis can provide novel insights into the molecular mechanisms, biomarkers, and therapeutic targets of DR. For example, HTS can reveal the expression profiles of genes, microRNAs, long noncoding RNAs, and circular RNAs in the retina or blood of patients with DR, and identify potential regulators of DR pathogenesis (Ye et al., 2016; Xu et al., 2022; Kanclerz and Tuuminen, 2023). HTS can also uncover the genetic variants, epigenetic modifications, and microbial communities that are associated with DR susceptibility or progression (Kowluru et al., 2015; Niu et al., 2021; Yang et al., 2021).

One of the emerging aspects of DR research is the role of immune cell infiltration in the retina. Immune cells, such as macrophages, T cells, B cells, and dendritic cells, are involved in the inflammatory response and vascular remodeling that occur during DR development (Farr et al., 2015). Immune cell infiltration can be influenced by various factors, such as hyperglycemia, oxidative stress, hypoxia, and cytokines (Hui et al., 2022). Moreover, immune cell infiltration can affect the function and survival of other retinal cell types, such as endothelial cells, pericytes, neurons, and glia (Meng et al., 2022). Therefore, understanding the immune landscape and its interaction with other components of the retina in DR is crucial for elucidating the pathophysiology and identifying new therapeutic strategies for this complex disease.

Ferroptosis is a type of programmed cell death that involves iron-dependent lipid peroxidation. It is different from other forms of cell death such as apoptosis, necrosis, and autophagy. Ferroptosis has been implicated in various diseases, including DR. FABP4 is highly expressed in diabetic complications, and inhibition of FABP4 slows the progression of diabetes-related diseases by ameliorating high-glucose-induced glomerular cell apoptosis and increasing serum insulin concentrations (Zhang et al., 2021). FABP4 inhibitor BMS309403 to enhance the activity of GPX4 and to reduce lipid peroxidation, which protects retinal cells in diabetic mice from oxidative stress (Shen et al., 2022).

The purpose of this research is to investigate the major biomarkers related with ferroptosis in DR, as well as their links to immune cell infiltration and immune pathway activation. We first extracted microarray datasets of DR patients and healthy controls from the GEO gene expression database for differentially expressed gene (DEG) analysis, and then used Weighted gene co-expression network analysis (WGCNA) to screen for co-expressed gene modules most relevant to the DR phenotype. Then, using machine learning approaches, we refined the screening of the six most important key genes and proved their diagnostic accuracy using ROC curves.

## 2 Materials and methods

### 2.1 Data acquisition and processing

The public GEO database was used to acquire gene expression data from DR patients. This study gathered two datasets (GSE60436, GSE102485). We standardize the quantile by “normalizeBetweenArrays” function, followed by a logarithmic transformation [e.g., “a” to “log (a +1)”] to conform to the requirements of a normal distribution for downstream data processing and analysis, and eliminate the batch effect by using the “combat” function in the “sva” R package (Leek, 2014) performs normalization, and log2 transformation is performed. Finally, a total of 21 DR patients and six normal controls were included.

### 2.2 Difference and enrichment analysis

The “limma” (Ritchie et al., 2015) package in R was used to screen DEGs for analysis (screening criterion |logFC|>1, adjusted *p* < 0.05). The R package “clusterProfiler” (Wu et al., 2021) was used to perform enrichment studies for Gene Ontology (GO) and Kyoto Encyclopedia of Genes and Genomes (KEGG).

### 2.3 Weighted gene co-expression network analysis

WGCNA has been widely applied to effectively investigate the relation between genome and clinical phenotype (Peng et al., 2022; Wang et al., 2023a; Chen et al., 2023). Gene co-expression matrices were created using the R software package “WGCNA” (Zhang and Horvath, 2005). According to the principle of scale-free network, scale-free co-expression network was created step by step by soft threshold (power = 15), and the neighbor-joining matrix was converted into topological overlapping matrix. Based on the topological overlap, the genes were clustered using the average chained hierarchical clustering method. According to the criteria of hybrid dynamic shear tree, the minimum number of genes in each gene network module was set to 60, the characteristic genes of each module were calculated, and the modules were clustered with the height set to 0.25. Modules related to the disease traits were identified with the highest correlation coefficients and the lowest *p*-values.

### 2.4 Screening and validation of diagnostic markers

Machine learning has been widely used in marker screening efforts (Le and Ou, 2016; Nguyen et al., 2024), we used multiple machine learning algorithms to screen for core genes. LASSO is a compression estimation method that creates a more accurate model by constructing a penalty function that forces it to compress certain regression coefficients or force the sum of the absolute values of the coefficients to be less than a fixed value, while setting certain coefficients to zero. LASSO regression analysis was performed on the crossover genes using the “glmnet” package in R (Friedman et al., 2010). We performed ten cross-validations and determined the value of λ based on the minimum criterion selection. SVM-RFE is a machine learning method based on Support Vector Machines for finding the best variables by removing the feature vectors generated by SVM. The SVM module was built to further characterise the diagnostic value of these biomarkers in BC through the e1071 package (Huang et al., 2014). Its jointly identified six genes were considered as candidate diagnostic markers. The ROC curve was used to determine the accuracy of the prediction.

### 2.5 Consensus clustering and immune infiltration analysis

Consensus Clustering is an unsupervised clustering method that is a common research method for classifying disease subtypes. SsGSEA (Hanzelmann et al., 2013) was used for the calculation of different immune cell scores in different classified populations.

### 2.6 Cell culture

Human retinal pigment epithelial cell line (ARPE-19) was purchased from the Procell Life Science & Technology in China (CL-0026) and cultured in DMEM/F12 medium containing 10% fetal bovine serum (FBS) (Gibco, United States). ARPE-19 was cultured in high glucose medium (30 mM anhydrous glucose) (HG) or normal glucose (5 mM) (NG) medium. High glucose-exposed cells were used as an *in vitro* model of diabetic retinopathy. Cultured at 37°C for 48 h.

### 2.7 Western blotting

Western blot was carried out according to the standard protocol and protein lysates were acquired from cultured cells. The primary antibodies used was PARP14 (Abcam, ab302710 at 1/1,000 dilution).

### 2.8 Cell transfection

The cDNA fragments of PARP14 and pcDNA3.1 vectors were prepared by EcoRV/XhoI double enzyme digestion. Then, PARP14 cDNA fragments were inserted into the pcDNA3. One vector. Afterwards, the PARP14 cDNA fragment was inserted into the pcDNA3.1 vector using T4 DNA ligase (Thermo Fisher Scientific, Waltham, MA, United States) to generate the pcDNA3.1/PARP14 overexpression vector. Small interfering RNA (siRNA) for PARP14 and control siRNA were purchased from Genomeditech (Shanghai, China). These recombinant plasmids were transfected using Lipofectamine 2000 (Invitrogen, Carlsbad, CA). The sequence for PARP14_si was as follows:

PARP14_si1: CCC​TCT​TGA​TGG​TGG​ATT​A.

PARP14_si2: GCA​CAG​CCT​TGT​ATG​GAA​A.

PARP14_si3: CAA​GCA​CCA​TTC​AAT​TAA​A.

### 2.9 Cell Counting Kit‐8 assay

Cell proliferation was measured using Cell Counting Kit-8 (Dojindo Molecular Technologies, Japan). Cells were seeded in 96-well plates for 48 h, then treated with 10 μL of CCK-8 solution for 4 h. An ELISA reader (Thermo Labsystems, Finland) was used to measure the absorbance at 450 nm.

### 2.10 Aqueous humor collection and qPCR analysis

Aqueous humor collection in diabetic retinopathy patients (DR group) and retina detachment patients (Control group), 20 cases in each group. Total RNA from the DR and control groups was extracted with TRIzol reagent (Ambion, Carlsbad, CA). RNA was reverse-transcribed into cDNA in strict accordance with the instructions of the reagent vendors, and then subjected to PCR reaction using the SYBR Green chimeric fluorescence method (all from vazyme China). Relative mRNA expression was assessed using the 2 ^−ΔΔCt^ method. All primers (Genepharma, Shanghai, China) were provided in Supplementary Table S1.

### 2.11 Statistical analysis

GraphPad Prism statistical software (version 8.0) and R (version 4.2.0) has been implemented for all analyses and graphs. To compare the two sets of data, the *t*-test or Wilcox test was utilized. The connection between putative diagnostic genes and immune cell infiltration was investigated using the Spearman rank correlation test. *p* < 0.05 was chosen as the statistical significance level.

## 3 Results

### 3.1 Identification of differential genes

**FIGURE 1:**
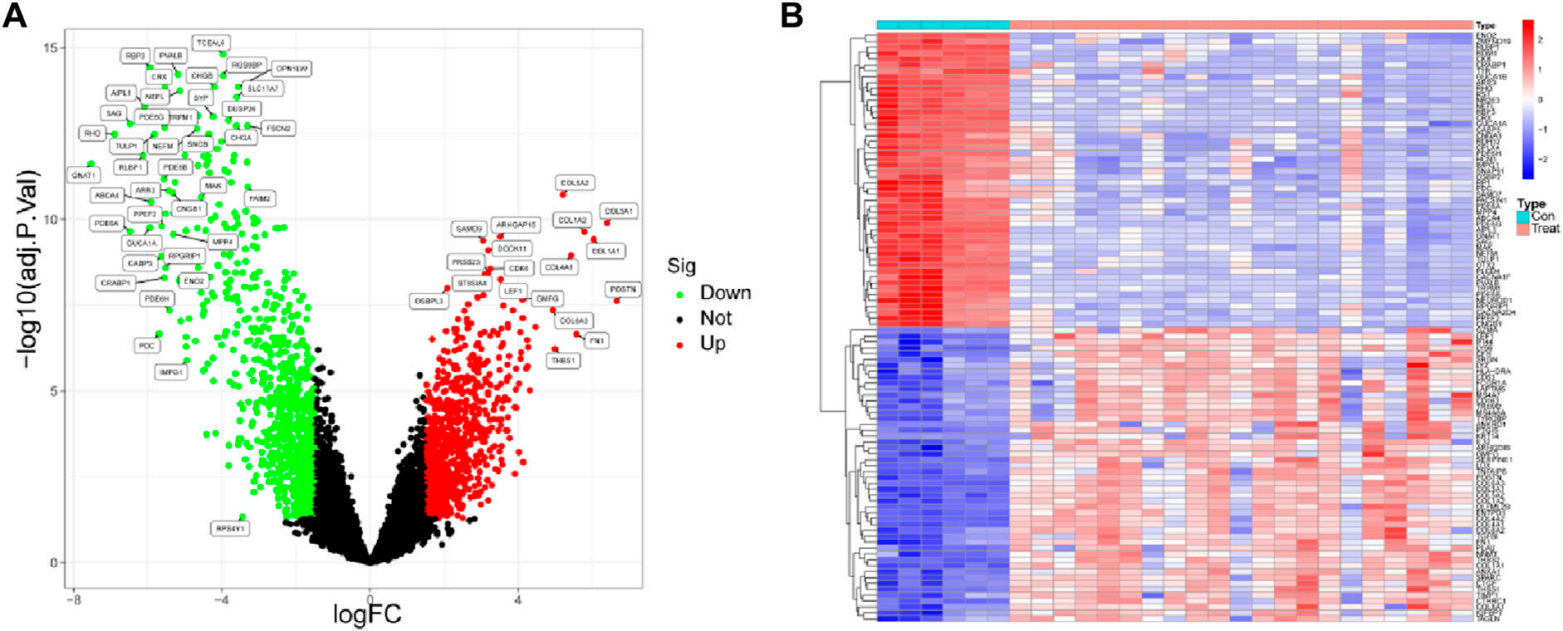
Identification of differentially expressed genes between DR samples and healthy samples. **(A)** Volcano plot and **(B)** heat map of differentially expressed genes.

The analysis of differential genes was performed in this study using the corrected matrix. Based on the criterion of |logFC|>1, a total of 2,897 DEGs were identified, of which 1,378 were upregulated genes and 1,518 were downregulated genes (Figure 1B). The most significantly altered genes were highlighted in Figure 1A.

### 3.2 WGCNA analysis identifies the most relevant modules for disease

**FIGURE 2:**
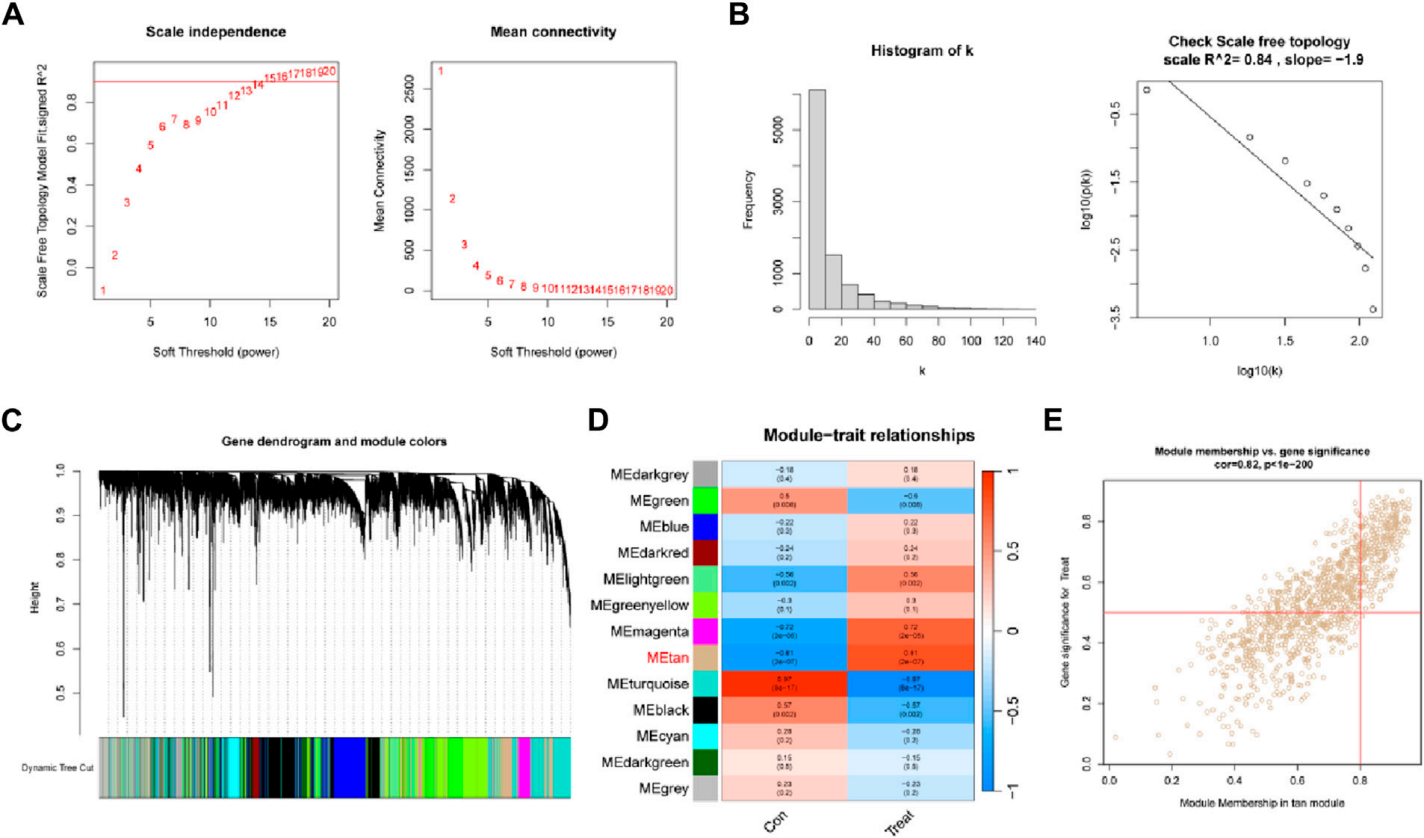
Weighted gene co-expression network analysis. **(A)** Soft threshold (power = 20) and scale-free topological fit index (R2 = 0.84). **(B)** Histogram of connectivity distribution. The scale-free topology is checked at a soft threshold of 15. **(C)** Gene hierarchical tree clustering diagram. **(D)** Heatmap showing the relationship between modules and DR functions. The values in the small cells in the plot represent the two calculated correlation values or coefficients between each feature and the feature values of each module, and the corresponding statistically significant *p*-values. **(E)** Scatter plot between gene significance (GS) and module membership (MM) in Tan.

We built a gene co-expression network to more precisely mine the core genes linked with the DR phenotype. The soft threshold was set at 15 to accommodate the network’s scale-free architecture (Figure 2A). To test whether the set parameter β satisfies the scale-free network, we first demonstrated the distribution of the connectivity of genes by histogram of k and calculated R2 equal to 0.84, which represents a good linear relationship (Figure 2B). A gene hierarchical clustering dendrogram was constructed by gene correlation and a total of 13 similar gene modules were identified (Figure 2C). Among them, the tan module had the highest positive correlation (*R* = 0.81) with DR (Figure 2D). By calculating the GS and MM values, we identified the most central disease genes in the modules (Figure 2E).

### 3.3 Identification of ferroptosis-associated DR genes

**FIGURE 3:**
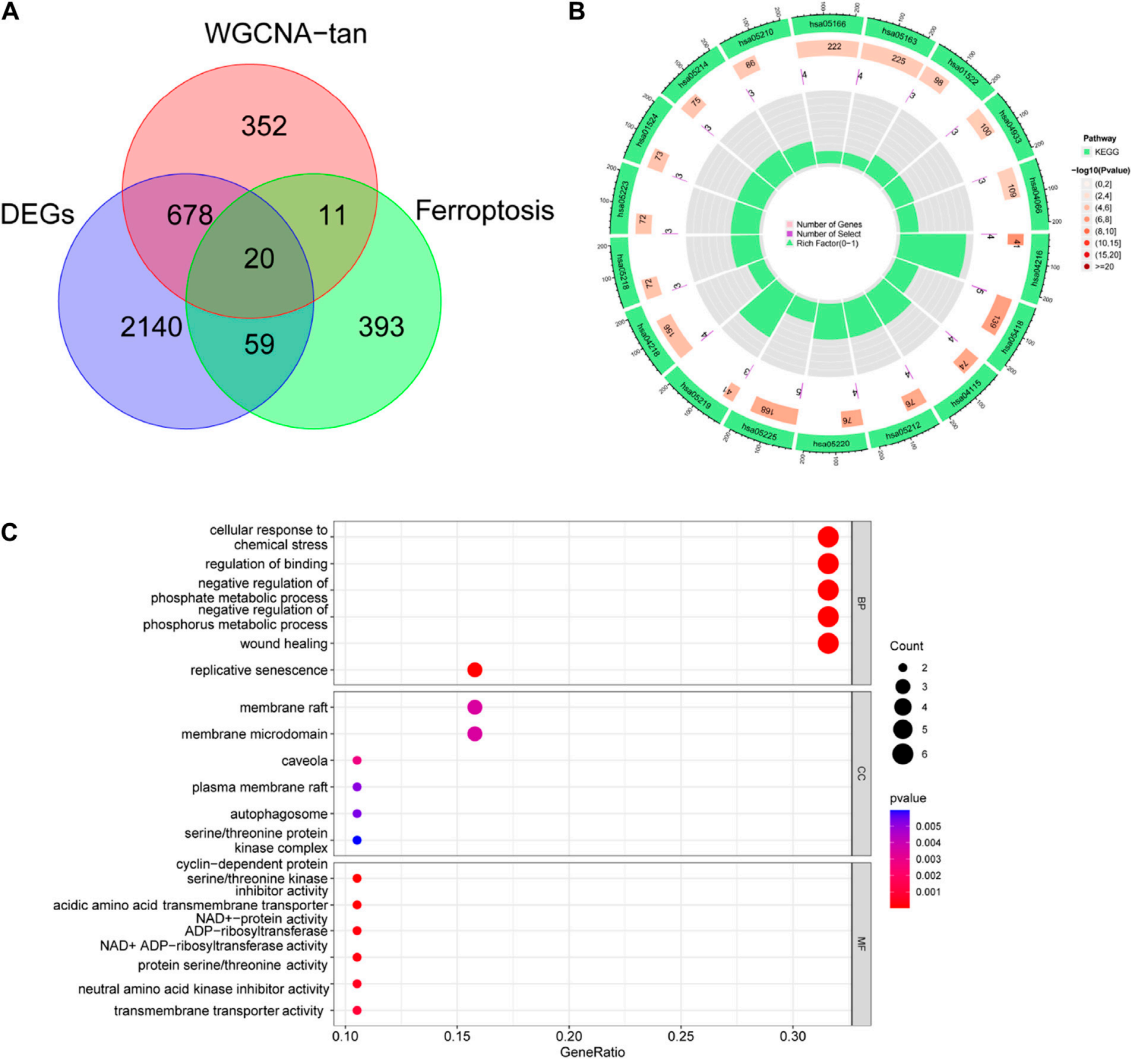
Identification of ferroptosis-associated DR genes. **(A)** Differential genes, ferroptosis-related genes and tan module genes jointly identified 20 ferroptosis related DR genes. **(B)** KEGG and **(C)** GO enrichment analysis of 20 genes.

Ferroptosis related genes were obtained from FerrDb (http://www.zhounan.org/ferrdb/current/). We took the intersection of the above DEGs with the significant genes and Ferroptosis related genes in the tan module and obtained 20 Ferroptosis related DR genes (Figure 3A). To determine the function of these genes, we performed GO, KEGG analysis. KEGG analysis identified significant enrichment in Ferroptosis, Fluid shear stress and atherosclerosis, p53 signaling pathway, Pancreatic cancer and Chronic myeloid leukemia (Figure 3B). GO analysis identified its molecular functions as cell cycle protein-dependent protein serine/threonine kinase inhibitor activity, acidic amino acid transmembrane transporter activity, and NAD + -protein ADP-ribosyl transferase activity (Figure 3C).

### 3.4 Machine learning-based identification of diagnostic markers

**FIGURE 4:**
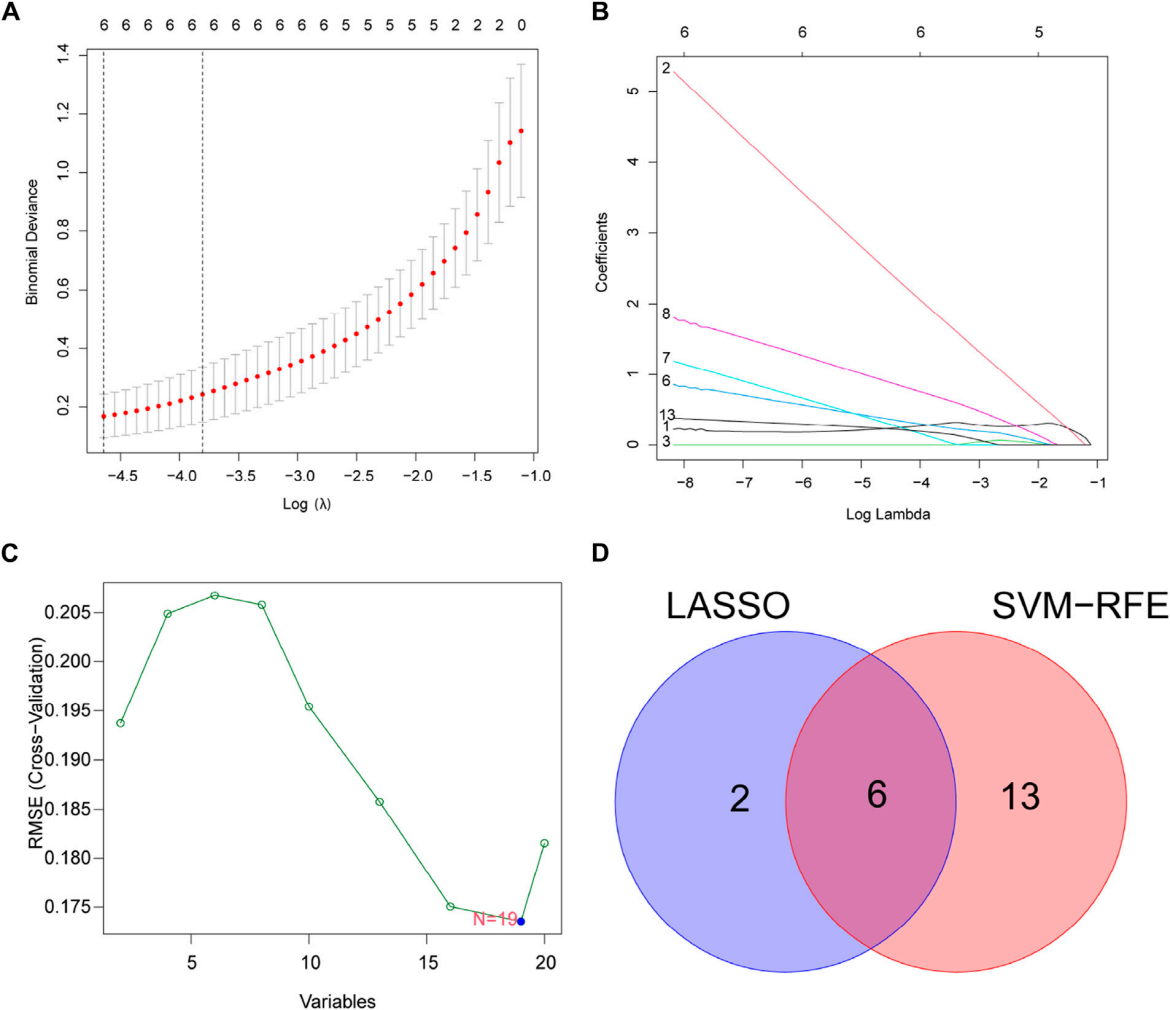
Select potential diagnostic biomarkers for DR. **(A,B)** Adjustment of feature selection in LASSO. **(C)** SVM-RFE technique for biomarker selection. **(D)** In this Venn diagram, SVM-RFE and LASSO algorithms show six diagnostic markers between them.

For finding potential biomarkers, the researchers utilized two separate algorithms. By using the LASSO regression algorithm, the genes were reduced to eight variables, which were identified as potential diagnostic biomarkers for DR (Figures 4A,B). The SVM-RFE method similarly reduced the genes to a subset of 19 genes (Figure 4C). Six overlapping features between these two algorithms (TMSB4X, NOX4, PARP14, SLC1A5, TP53, and CDKN2A) were finally selected as markers of DR (Figure 4D).

### 3.5 Validation of expression and diagnostic performance and functional analysis of core diagnostic genes

**FIGURE 5:**
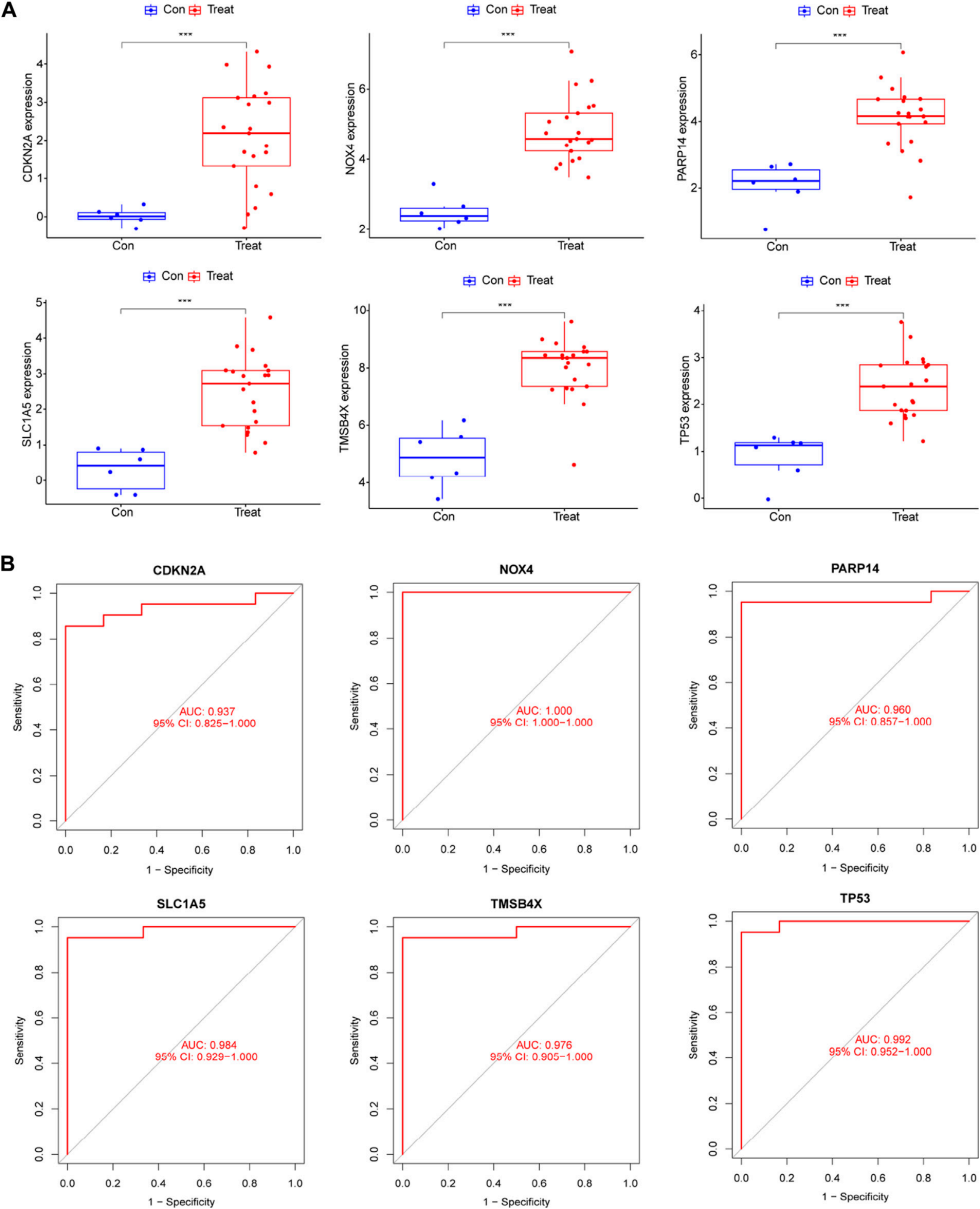
The expression levels and diagnostic performance of six core diagnostic genes in DR. **(A)** Expression of six core diagnostic genes in DR. ROC curve to detect diagnostic performance including **(B)** TMSB4X, NOX4, PARP14, SLC1A5, TP53, and CDKN2A.

**FIGURE 6:**
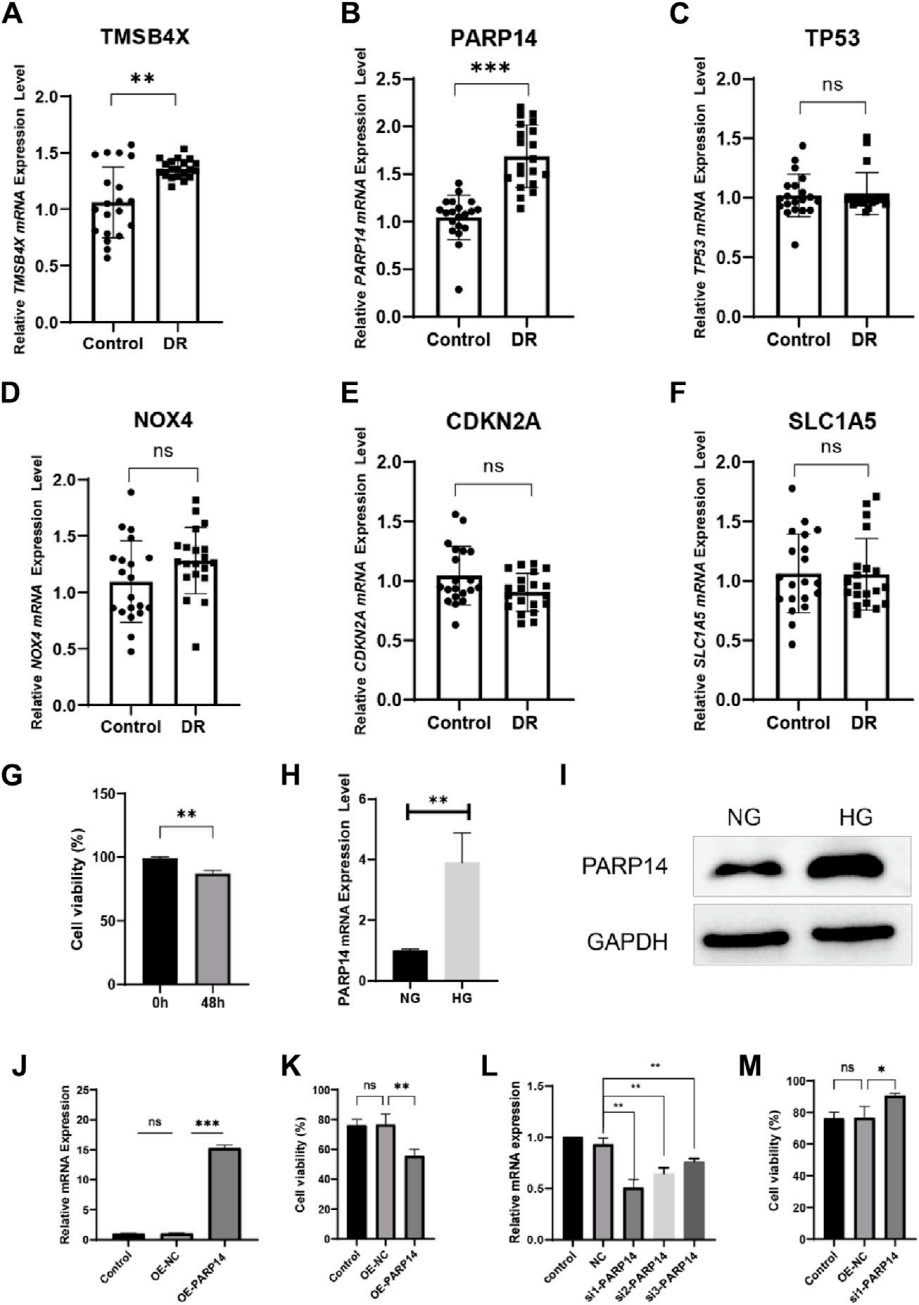
Validate the expression and function of core diagnostic genes. Aqueous humor collection in diabetic retinopathy patients (DR group) and retina detachment patients (Control group) was used to verify mRNA level expression of **(A)** TMSB4X, **(B)** PARP14, **(C)** TP53, **(D)** NOX4, **(E)** CDKN2A, **(F)** SLC1A5. **(G)** Activity of ARPE-19 cells after 48 h in high glucose culture. PARP14 **(H)** mRNA and **(I)** protein levels after 48 h of high glucose culture. **(J)** Validation of mRNA levels after overexpression of PARP14 cells. **(K)** Overexpression of PARP14 under high glucose inhibited the survival of ARPE-19 cells even more. **(L)** Validation of mRNA levels after knockdown of PARP14 cells. **(M)** Knockdown of PARP14 inhibits ARPE-19 cell activity.

We compared the expression of six genes in DR and all genes were upregulated in the DR group (all *p* < 0.05; Figure 5A). Its accuracy as a diagnostic marker was assessed by the area under the ROC curve. The AUC value of NOX4 was 1, and the AUC values of the other five genes were all above 0.9, which indicates its great potential as a diagnostic marker (Figure 5B). We examined the expression of six genes in aqueous humor in diabetic retinopathy patients and retina detachment patients, Interestingly, the expression of TMSBX and PARP14 was upregulated in DR groups (Figures 6A,B), while the levels of the other four core mRNAs remained unchanged (Figures 6C–F). Since PARP14 was significantly upregulated in the DR, we used PARP14 for subsequent target validation. Under high glucose culture, ARPE-19 cells started to apoptose after 48 h (*p* < 0.05; Figure 6G). Meanwhile, PARP14 mRNA and protein levels were significantly increased (*p* < 0.05; Figures 6H,I). To further clarify the cellular function of PARP14, we constructed overexpression and knockdown cell lines (Figure 6J,L). The results showed that overexpression of PARP14 more inhibited retinal cell survival under the effect of high glucose (*p* < 0.05; Figure 6K), and, in contrast, knockdown of PARP14 inhibited apoptosis of retinal cells (*p* < 0.05; Figure 6M).

### 3.6 The link between immune cell infiltration and core diagnostic genes

**FIGURE 7:**
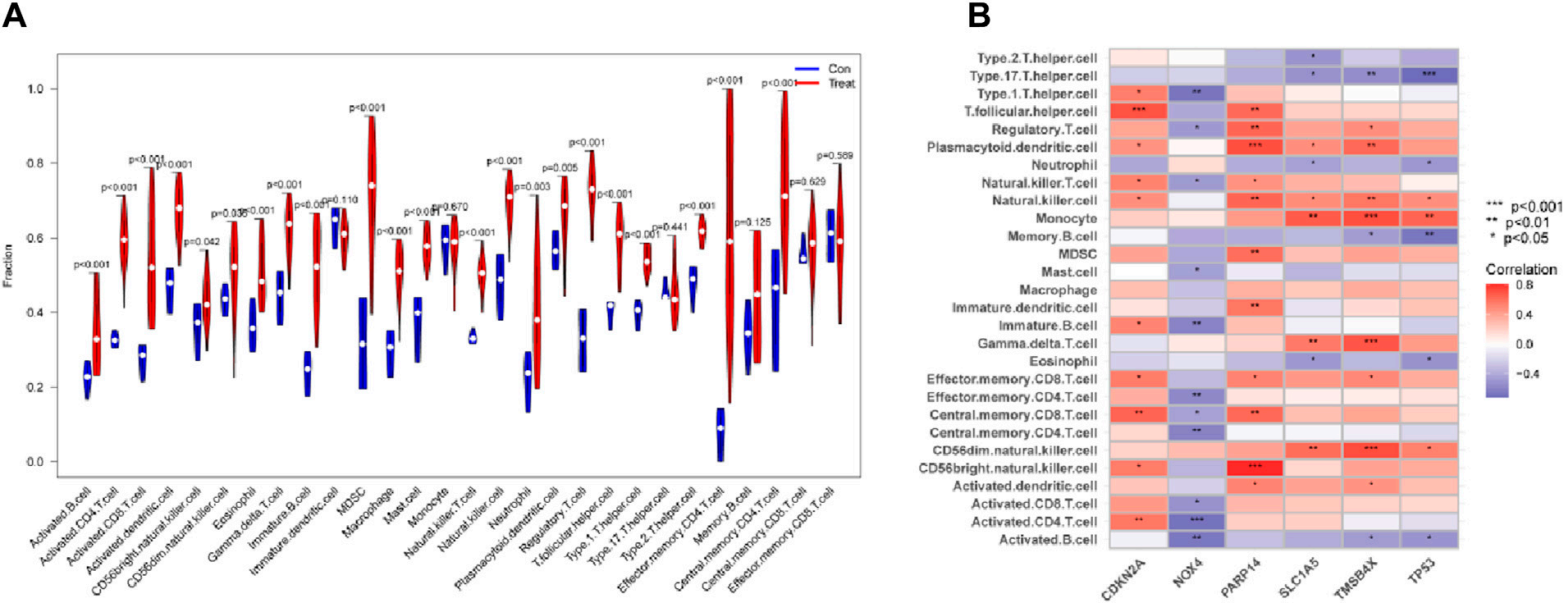
Link between six genes and immune cells. **(A)** The relationship between Immune cells DR and normal samples. **(B)** Correlations between six genes and immune cells in DR.

SsGSEA was put to use to explore differences in immune infiltration expression between DR patients and healthy controls. Immune cells were shown to be significantly active in DR patients, with enhanced infiltration of activated CD4^+^ T cells, activated CD8^+^ T cells, macrophages, mast cells, and NK cells (Figure 7A). NOX4 was found to be negatively connected with central memory CD4 T cells, central memory CD8 T cells, and effector memory CD4^+^ T cells engaged in adaptive immunological responses (all *p* < 0.05) in a correlation study. CDKN2A and PARP14, on the other hand, were positively associated with a number of immune cells, including CD56 bright natural killer cells and central memory CD8^+^ T cells (all *p* < 0.01) (Figure 7B). These findings add to the body of evidence suggesting a cellular-molecular mechanism by which ferroptosis regulates DR progression.

### 3.7 Molecular classification of DR disease

**FIGURE 8:**
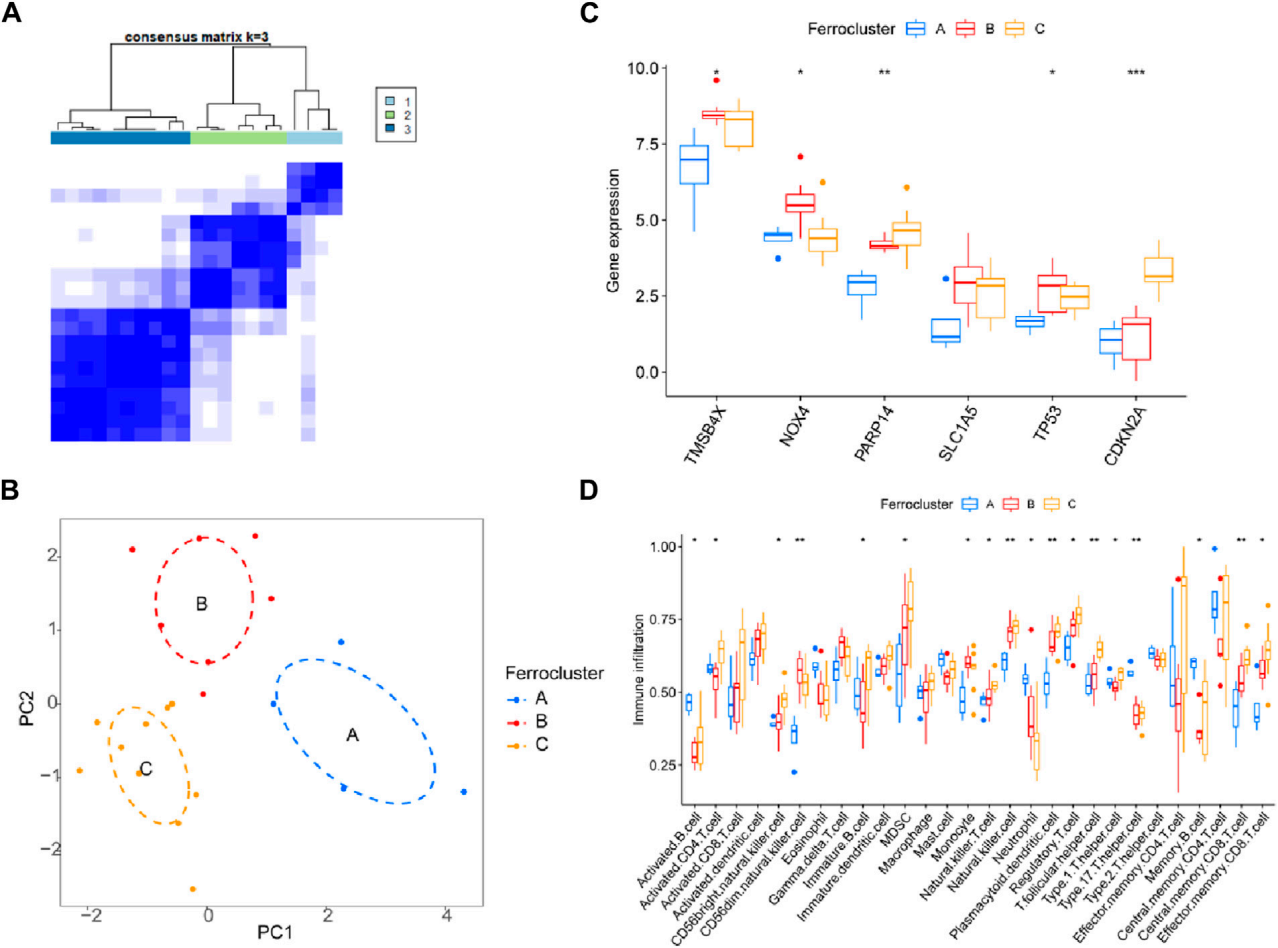
Molecular classification of DR disease. **(A)** Unsupervised clustering divides DR patients into 3 clusters. **(B)** PCA analysis. **(C)** Expression of six genes and **(D)** immune cell subsets in three clusters.

Previous studies have shown that ferroptosis-related genes can serve as molecular typing for a variety of diseases, and to determine whether the six core genes we identified could serve as molecular features for disease typing. We used these six genes to successfully classify DR patients into three types (named C-A, C-B, C-C) by unsupervised clustering (Figure 8A). PCA analysis confirmed that the three populations have different expression distributions (Figure 8B). The six core molecules were least expressed in population C-A, NOX4 was highest in population C-B, and CDKN2A was highest in population C-C (Figure 8C). There was an increasing trend in the scoring of immune cells, including MDSC, NK cells, Tregs, central memory CD8^+^ T cells and effector memory CD8^+^ T cells (*p* < 0.05; Figure 8D).

## 4 Discussion

DR is a common complication of diabetes that affects the retina, which cause vision loss and blindness due to damage to the blood vessels and neurons in the retina. Ferroptosis has been implicated in various diseases, such as cancer, neurodegeneration, and ischemia-reperfusion injury. However, the role of ferroptosis in DR is less studied and not fully understood. A previous review described that ferroptosis may be involved in the pathogenesis of DR by inducing inflammation, vascular leakage, ischemia, and neovascularization in the retina (He et al., 2023). Therefore, studying ferroptosis in DR may provide insights into the molecular mechanisms and therapeutic targets for preventing or reversing retinal damage.

This study provides the first comprehensive survey of the evidence for the possible involvement of ferroptosis in the presence of DR and culminates in the identification of three key genes (TMSB4X, NOX4, PARP14, SLC1A5, TP53, and CDKN2A) that play a critical regulatory role and could serve as potential biomarkers for the diagnosis of DR. TMSB4X is an actin-isolating protein that plays an important role in the organization of the cytoskeleton. Numerous studies have demonstrated the effects of Tβ4 on cell migration, proliferation, apoptosis and inflammation after exogenous treatment (Kimura et al., 1990). Naoki Orii’s construction of Wiki-Pi, a web-based database of human PPIs, postulates that IGSF21 mediates DR by interacting with TMSB4X and is involved in the regulation of cellular responses to external stimuli, cytoskeletal organization, and molecular activity (Orii and Ganapathiraju, 2012). NOX4, which we identified, has been widely recognized as a novel therapeutic target for diabetic vascular complications (Wang et al., 2023b). NOX4 promotes vascular permeability and neovascularization in retinopathy (Deliyanti et al., 2020). Topically administered NOX4 inhibitor GLX7013114 is effective in treating early pathological events in DR (Dionysopoulou et al., 2023). Whereas previous articles have reported that NOX4 activation regulates ferroptosis through astrocytic mitochondrial dysfunction (Boonpraman et al., 2023). NOX4-triggered osteoblastic ferroptosis in osteoporotic bone loss driven by excess iron accumulation (Zhang et al., 2023). These results suggest that NOX4 is greatly likely to affect DR through ferroptosis. Diabetic retinas show increased TP53 transcription, which is consistent with our study (Mohammad et al., 2019). The study by Liu *et al.* also identified TP53 as a potential diagnostic marker for DR (Liu et al., 2022). The SLC1A5 gene encodes a sodium-dependent neutral amino acid transporter. Zhou *et al.* study confirmed that a novel miR-338-3p/SLC1A5 axis reprograms the retinal pigment epithelium to enhance its resistance to high glucose-induced cellular ferroptosis (Zhou et al., 2022). On the other hand, we found in our enrichment analysis that these genes are involved in Acidic Amino Acid Transmembrane Transporter Activity, which affects the cellular environment and metabolism. In diabetic retinopathy, accumulation of advanced glycation end products (AGEs) and altered amino acid metabolism affect cellular function and induce inflammation, leading to disease pathogenesis (Chen et al., 2013). NAD ± Protein ADP-Ribosyltransferase Activity is the process of transferring ADP ribose from NAD + to target proteins, a process known as ADP-ribosylation. This modification regulates a variety of cellular processes, including DNA repair, transcription, and metabolism, and affects glucose metabolism and cellular responses to stress in the DR (Hopp et al., 2019; Chen et al., 2021). Therefore, further study of DR-associated ferroptosis genes is significant in elucidating the pathogenesis of DR.

Biomarkers contribute to the understanding of DR and help develop new treatments or new clinical strategies to prevent vision loss (Jenkins et al., 2015). A large number of studies have now provided preliminary insights into a variety of key target molecules in DR-related progression, e.g., oxidative stress, apoptosis, inflammation, etc (Lopez-Contreras et al., 2020). The ocular surface is frequently exposed to intense light and solar UV radiation, and high metabolic activity also increases the production of ROS and OS. This is a common adaptation secondary to inflammation and diabetes, which produce more ROS media at the ocular surface (Tamhane et al., 2019). Therefore, adequate levels of antioxidant enzymes are essential for oxidative and reductive (redox) homeostasis (Kulaksizoglu and Karalezli, 2016), which includes the key glutathione peroxidases (GPX family) that control peroxide concentrations in the lipid layer and protect the ocular surface from OS damage (Nezzar et al., 2007). Whereas GPX4 is a central regulator of ferroptosis, GPX4 inhibitors are potent inducers of ferroptosis (Singh et al., 2022). In addition to ferroptosis, apoptosis is the most studied type of cell death in diabetic retinopathy. Apoptosis of retinal capillary cells may contribute to capillary “detachment” and retinal ischemia in DR. There is therefore great interest in caspases that may be involved in initiating and executing this apoptotic process, especially Caspase-3, which induces an immune response after apoptosis (Mohr et al., 2002), and inhibition of Caspase-3 reduces retinal cell apoptosis. Nuclear factor-k (NF-k) is an important polyphenolic nuclear factor involved in apoptosis and cellular neogenesis, and it can be activated by a variety of signals, such as IL-1, TNF-, and OS. Several studies have shown that it is closely associated with inflammation, tumor emergence, apoptosis, and other pathological processes. However, the major pathologic changes in DR include retinal apoptosis and neovascularization. This highlights the role of inflammatory factors in DR (Jiang et al., 2015; Forrester et al., 2020).

According to some research articles, there are different types of immune cells that are involved in DR, such as macrophages, microglia, T cells, B cells, and neutrophils. These immune cells can produce various inflammatory molecules, such as cytokines, chemokines, and adhesion molecules, that can affect the function and survival of retinal cells. For example, some studies have shown that macrophages and microglia, which are the resident immune cells of the retina, can release pro-inflammatory cytokines such as IL-1β, IL-6, TNF-α, and IL-18, which can induce vascular leakage, ischemia, and neovascularization in DR (Semeraro et al., 2015; Duh et al., 2017). T cells and B cells, which are adaptive immune cells that can recognize specific antigens, can also infiltrate the retina and produce cytokines and antibodies that can modulate the inflammatory response and affect the vascular integrity (Yue et al., 2022). Our work identifies upregulation of B and T cells in the DR, which may be related to CDKN2A and PARP14.

In addition, our work identified six ferroptosis-associated DR genes that can be used as molecular typing to distinguish DR patients into different molecular subtypes. These patient stratifications may be relevant for ferroptosis-related targeted therapy and precise treatment selection.

Although the current study has mostly realized our initial vision, there are still some shortcomings. Firstly, due to the sample limitations of the publicly available dataset collection, more independent cohorts are needed for validation despite our initial experimental validation. In addition, the relationship between core molecules and DR remains unelucidated, and further biochemical experiments are needed to follow up to elucidate the critical role of ferroptosis in DR. Finally, although immune infiltration analysis and correlation with diagnostic genes were performed in DR patients, subsequent clinical trials and basic science experiments are needed to validate the results of the bioinformatics analysis.

In conclusion, we comprehensively summarized the core molecular features of ferroptosis-related genes in DR and can be used as a diagnosis for DR patients. Further, we also analyzed the effect of these core genes on the immune infiltration status of DR patients, which is important for studying the role of inflammation in the immune system, the molecular mechanisms, the pathogenic effects, and the treatment of DR.

## 5 Conclusion

In conclusion, we analyzed for the first time the potential link of ferroptosis in the pathogenesis of DR. We revealed ferroptosis-related genes (TMSB4X, NOX4, PARP14, SLC1A5, TP53, and CDKN2A) as diagnostic markers of DR, and preliminarily explored the links with the DR immune microenvironment and molecular typing of the disease.

## Data Availability

The original contributions presented in the study are included in the article/Supplementary Material further inquiries can be directed to the corresponding author.
