# Supplementary material for: Identification of biomarkers associated with ferroptosis in diabetic retinopathy based on WGCNA and machine learning
Source: Front Genet. 2024 May 28;15:1376771. doi: 10.3389/fgene.2024.1376771 (PMC11165058; doi:10.3389/fgene.2024.1376771)
Supplement: Supplementary file 1 [file Table1.DOCX]

Table S1:The primer sequences used in this study.

| Gene | Forward(5'->3') | Reverse(5'->3') |
| --- | --- | --- |
| TMSB4X | CTTCGCTTTTCCTCCGCAAC | CGGCCTTCGTTGTCAGTAGT |
| NOX4 | CAGTCTTTGACCCTCGGTCC | TGATCCTCGGAGGTAAGCCA |
| PARP14 | TCCAGGTGGCTTCTGGAGAT | TGCTGAGCTTGCTGAGAACA |
| SLC1A5 | ACAGGATATTGAGGGGAGGCT | GGATGAAACGGCTGATGTGC |
| TP53 | AAGTCTAGAGCCACCGTCCA | ACCATCGCTATCTGAGCAGC |
| CDKN2A | CCGAATAGTTACGGTCGGAGG | AATCGGGGATGTCTGAGGGA |
| GAPDH | AATGGGCAGCCGTTAGGAAA | GCGCCCAATACGACCAAATC |
